# Human Milk Oligosaccharides Reduce Murine Group B *Streptococcus* Vaginal Colonization with Minimal Impact on the Vaginal Microbiota

**DOI:** 10.1128/msphere.00885-21

**Published:** 2022-01-05

**Authors:** Marlyd E. Mejia, Samantha Ottinger, Alison Vrbanac, Priyanka Babu, Jacob J. Zulk, David Moorshead, Lars Bode, Victor Nizet, Kathryn A. Patras

**Affiliations:** a Department of Molecular Virology and Microbiology, Baylor College of Medicine, Houston, Texas, USA; b Department of Pediatrics, UC San Diego, La Jolla, California, USA; c Larsson-Rosenquist Foundation Mother-Milk-Infant Center of Research Excellence, UC San Diego, La Jolla, California, USA; d Skaggs School of Pharmacy and Pharmaceutical Sciences, UC San Diego, La Jolla, California, USA; e Alkek Center for Metagenomics and Microbiome Research, Baylor College of Medicine, Houston, Texas, USA; University of Nebraska Medical Center

**Keywords:** antimicrobial activity, group B *Streptococcus*, human milk oligosaccharides, vaginal colonization, vaginal microbiota

## Abstract

Group B Streptococcus (GBS) colonizes the vaginal mucosa of a significant percentage of healthy women and is a leading cause of neonatal bacterial infections. Currently, pregnant women are screened in the last month of pregnancy, and GBS-positive women are given antibiotics during parturition to prevent bacterial transmission to the neonate. Recently, human milk oligosaccharides (HMOs) isolated from breastmilk were found to inhibit GBS growth and biofilm formation *in vitro*, and women that make certain HMOs are less likely to be vaginally colonized with GBS. Using *in vitro* human vaginal epithelial cells and a murine vaginal colonization model, we tested the impact of HMO treatment on GBS burdens and the composition of the endogenous microbiota by 16S rRNA amplicon sequencing. HMO treatment reduced GBS vaginal burdens *in vivo* with minimal alterations to the vaginal microbiota. HMOs displayed potent inhibitory activity against GBS *in vitro*, but HMO pretreatment did not alter adherence of GBS or the probiotic Lactobacillus rhamnosus to human vaginal epithelial cells. In addition, disruption of a putative GBS glycosyltransferase (Δ*san*_0913) rendered the bacterium largely resistant to HMO inhibition *in vitro* and *in vivo* but did not compromise its adherence, colonization, or biofilm formation in the absence of HMOs. We conclude that HMOs are a promising therapeutic bioactive to limit GBS vaginal colonization with minimal impacts on the vaginal microenvironment.

**IMPORTANCE** During pregnancy, GBS ascension into the uterus can cause fetal infection or preterm birth. In addition, GBS exposure during labor creates a risk of serious disease in the vulnerable newborn and mother postpartum. Current recommended prophylaxis consists of administering broad-spectrum antibiotics to GBS-positive mothers during labor. Although antibiotics have significantly reduced GBS neonatal disease, there are several unintended consequences, including altered neonatal gut bacteria and increased risk for other types of infection. Innovative preventions displaying more targeted antimicrobial activity, while leaving the maternal microbiota intact, are thus appealing. Using a mouse model, we found that human milk oligosaccharides (HMOs) reduce GBS burdens without perturbing the vaginal microbiota. We conclude that HMOs are a promising alternative to antibiotics to reduce GBS neonatal disease.

## INTRODUCTION

Group B Streptococcus (GBS or Streptococcus agalactiae) is a Gram-positive bacterium that colonizes the gastrointestinal and vaginal tracts of ∼18% of pregnant women globally (1), exposing ∼20 million infants to GBS at, or prior to, delivery (2). The majority of children born to GBS-positive women themselves become colonized without symptoms (3); however, a subset of these infants (>300,000 annually) develop invasive GBS infections, accounting for upwards of 100,000 infant deaths each year worldwide (2). In addition, 57,000 annual stillbirths are attributed to GBS infections (2), though this may be an underestimate since GBS is also the most frequently cultured bacterium in midgestation spontaneous abortions (4). Because maternal colonization is a risk factor for neonatal infections, universal screening in late pregnancy and intrapartum antibiotic prophylaxis (IAP) to GBS-positive or at-risk mothers is the current standard of care in many countries. These preventative measures have decreased, but not eradicated, GBS early-onset disease (5). However, this early antibiotic exposure disrupts the infant microbiota, and the potential adverse consequences of this perturbation are not fully established (6–10).

Breastfeeding has long been associated with improved infant health, reduced risk of infectious disease, and accelerated immune and microbial maturation within the gut (11–13). Human milk oligosaccharides (HMOs), the third most abundant component of breastmilk, are a group of structurally complex, unconjugated glycans that are recalcitrant to host digestive enzymes. HMOs provide nutritional advantage for beneficial microbes in the infant gut and drive immune maturation at the gut epithelium (13–16). Moreover, HMOs may protect against neonatal pathogens by acting as soluble “decoy” receptors for enteric pathogens (17, 18), through neutralization of bacterial toxins (19, 20), or via direct antimicrobial activity, including against GBS (21–24). Although the mechanism of HMO-mediated GBS inhibition is not known, GBS expression of a putative glycosyltransferase (locus *san*_0913) is necessary for inhibitory activity (21), and HMO exposure lowers GBS sensitivity to antibiotics, including vancomycin, erythromycin, and trimethoprim (21, 25, 26). Additional support for HMO-mediated anti-GBS activity stems from clinical observations that mothers who produce a functional variant of the fucosyltransferase enzyme FUT3, which attaches fucose in an α1-3 or α1-4 linkage to form certain HMOs, are less likely to be vaginally colonized by GBS (27).

We hypothesized that HMOs may reduce GBS vaginal colonization *in vivo* either through direct antimicrobial activity, or through indirect activity on the vaginal epithelium and/or vaginal microbiota. Here, we test this hypothesis using a murine model of GBS vaginal colonization and pooled HMOs (pHMOs) isolated from human breastmilk. We further assess the impact of pHMOs on bacterial attachment to human vaginal epithelial cells and phenotypically characterize a GBS strain that is resistant to HMO inhibition (21). Combined, our findings support the continued exploration of HMOs as a therapeutic strategy for GBS in pregnancy and the neonatal period.

## RESULTS

### Topical pHMO treatment reduces GBS vaginal burdens *in vivo*.

**FIG 1:**
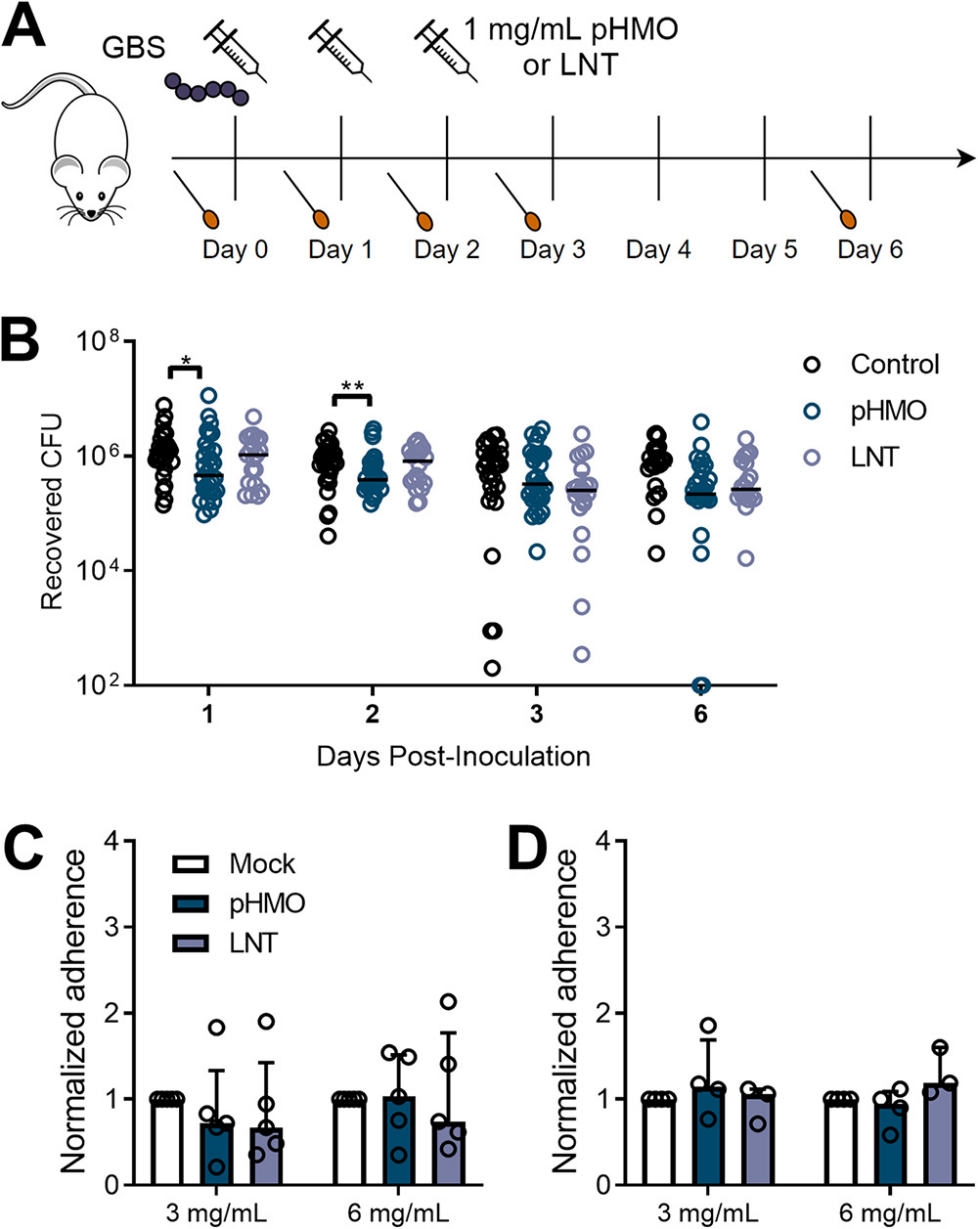
Treatment with pHMOs, but not a specific HMO LNT, reduces GBS vaginal burdens in mice and does not impact adherence to human vaginal epithelial cells. (A) Experimental timeline for the GBS colonization model. Baseline vaginal swabs were collected on day 0 prior to GBS inoculation with 1 × 10^7^ CFU of GBS COH1. Mice were treated with 1 mg of pHMOs or lacto-N-tetraose (LNT) 2 h postinfection and on the two subsequent days. Mice were swabbed prior to daily treatment with HMOs, as well as 1 and 4 days after the last HMO treatment. Mouse and syringe images are available open source through pixabay. (B) GBS burdens recovered from mouse vaginal swabs over the 6-day time course (*n *= 20 to 30/group). (C and D) Adherence of GBS COH1 (C) or Lactobacillus rhamnosus GG (D) to VK2 cells pretreated with pHMOs or LNT for 18 h. Adherence was normalized to mock-treated controls. Symbols represent individual mice (B) or the means of four to five independent experimental replicates (C and D), with lines representing medians and interquartile ranges. Data were analyzed by using the Kruskal-Wallis with Dunn’s multiple-comparison test (B) or two-way ANOVA with Dunnett’s multiple-comparison test (C and D). **, *P < *0.01; *, *P < *0.05. All other comparisons were not significant.

To determine the effect of HMOs on GBS vaginal colonization *in vivo*, wild-type C57BL/6J female mice were vaginally inoculated with GBS COH1, a serotype III ST17 neonatal sepsis clinical isolate (28). Mice were treated with pHMOs (1 mg/dose) 2 h after GBS inoculation, and dosing was repeated on the following two consecutive days. Lacto-N-tetraose (LNT), a commercially produced HMO that inhibits GBS growth *in vitro* (21) was included as a treatment condition to test the efficacy of a single HMO. Vaginal swabs were collected prior to pHMO treatment on days 0, 1, and 2, as well as on days 3 and 6 postinoculation (Fig. 1A). Treatment with pHMOs significantly reduced GBS vaginal burdens on day 1 (*P = *0.023) and 2 (*P = *0.009) during active treatment, but these differences were resolved at day 3 and 6 after pHMO treatment ceased (Fig. 1B). No differences between LNT and mock-treated groups were observed at any time point. In addition, endogenous vaginal *Enterococcus* spp. were distinguished on the Streptococcus selective media, but no differences between treatment groups were detected (see Fig. S1 in the supplemental material).

10.1128/msphere.00885-21.2 FIG S1 Treatment with pHMOs or LNT does not reduce endogenous *Enterococcus* spp. vaginal burdens in mice. As described in Fig. 1, baseline vaginal swabs were collected on day 0 prior to GBS inoculation with 1 × 10^7^ CFU of GBS COH1. Mice were treated with 1 mg of pHMOs or lacto-N-tetraose (LNT) at 2 h postinfection and on the two subsequent days. Mice were swabbed prior to daily treatment with HMOs, as well as 1 and 4 days after the last HMO treatment. *Enterococcus* sp. burdens recovered from mouse vaginal swabs over the 6-day time course are shown. Mice that did not culture *Enterococcus* at any time point were excluded (*n* = 7 to 22/group). Data were analyzed by Kruskal-Wallis with Dunn’s multiple-comparison test, and all comparisons are not significant. Download FIG S1, TIF file, 0.2 MB. Copyright © 2022 Mejia et al. 2022 Mejia et al. https://creativecommons.org/licenses/by/4.0/ This content is distributed under the terms of the Creative Commons Attribution 4.0 International license.

### Vaginal epithelial HMO exposure does not impact adherence of GBS or probiotic *Lactobacillus*.

Because HMOs can reduce pathogen adherence (29–31) and promote adherence of beneficial bacteria to the host epithelium (32), we tested the impact of epithelial HMO pretreatment on adherence of GBS or the probiotic Lactobacillus rhamnosus GG to human vaginal epithelial (VK2) cells. We observed no effect of pHMO or LNT pretreatment on GBS adherence to VK2 cells at two different concentrations (Fig. 1C), nor did HMO pretreatment alter L. rhamnosus adherence to VK2 cells (Fig. 1D).

### HMO resistance conferred by disruption of *san*_0913 does not alter GBS biofilm formation, adherence, susceptibility to antibiotics, or *in vivo* colonization in the absence of HMOs.

**FIG 2:**
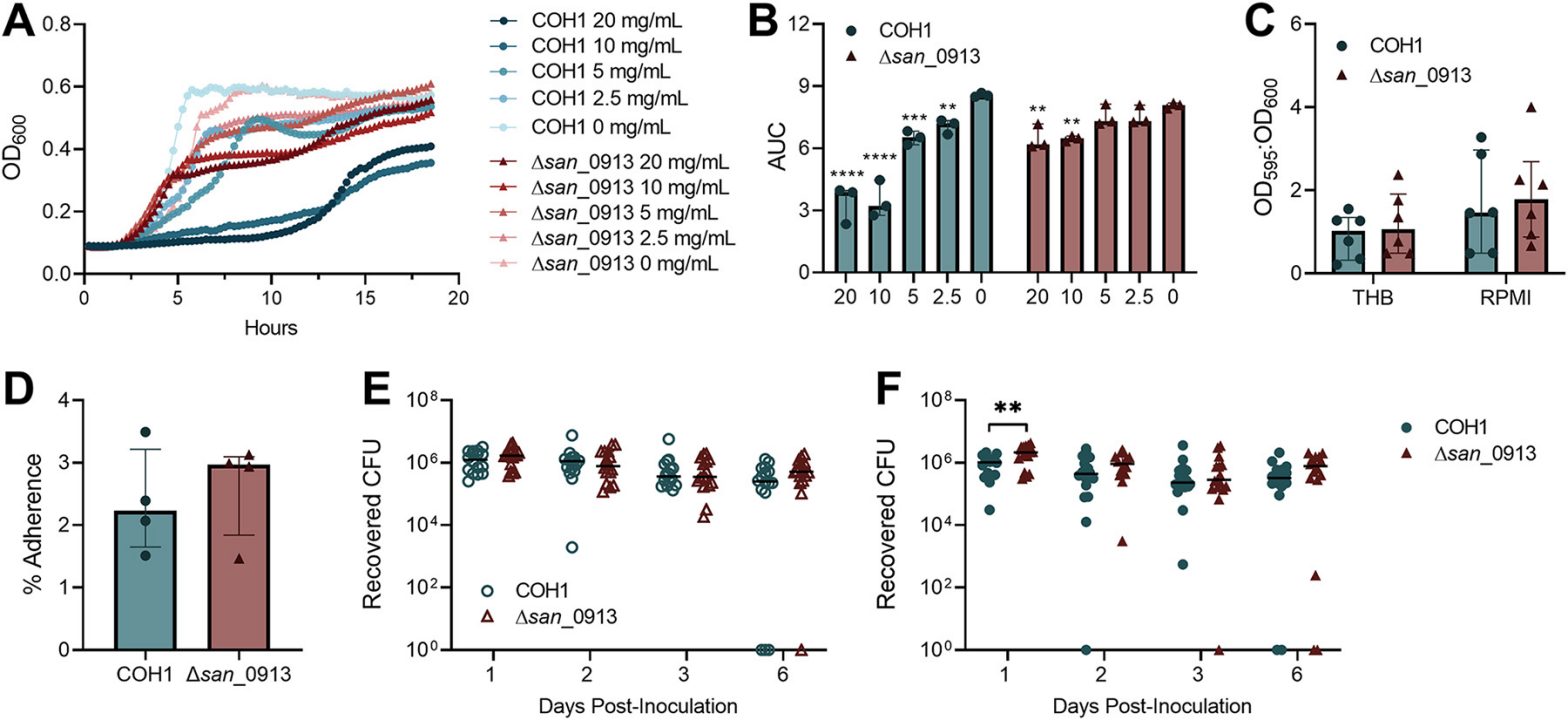
HMO resistance conferred by disruption of *san*_0913 does not alter GBS biofilms, adherence, or *in vivo* colonization in the absence of HMOs. (A) Growth curves of WT COH1 and Δ*san*_0913 in RPMI 1640 supplemented with 0, 2.5, 5, 10, or 20 mg/mL pHMOs and cultured for 18 h, as measured based on the optical density (OD_600_). (B) Area-under-the-curve analysis of growth curves from (A). Comparisons shown are to 0-mg/mL pHMO controls. (C) Biofilm formation of COH1 and Δ*san*_0913 in THB or RPMI 1640 quantified by crystal violet staining and expressed as a ratio of crystal violet absorbance versus the total bacterial biomass (OD_595_ to OD_600_). (D) Percent adherence of COH1 and Δ*san*_0913 to VK2 cells after 30 min of infection (MOI = 1). (E) Mice were vaginally inoculated with 1 × 10^7^ CFU of COH1 or Δ*san*_0913 and then vaginally swabbed at the indicated time points. Recovered GBS CFU recovered from swabs are shown. (F) Mice were inoculated as in panel E and treated with pHMOs as indicated in Fig. 1A. The results for the recovered GBS CFU recovered from swabs are shown. Symbols represent the median of three independent experiments (A), the means of three to six independent experiments (B to D), or individual mice from two combined independent experiments (*n *=* *16/group [E and F]). Lines indicate median values and interquartile ranges. Data were analyzed by two-way repeated measures ANOVA with Dunnett’s multiple-comparison test (B), two-way ANOVA with Sidak’s multiple-comparison test (C), Wilcoxon matched-pairs signed rank test (D), or two-stage Mann-Whitney test (E and F). ****, *P < *0.0001; ***, *P < *0.001; **, *P < *0.01; *, *P < *0.05. All other comparisons are not significant.

Although the exact mechanism of HMO anti-GBS activity has yet to be established, increased GBS sensitivity to intracellular targeting antibiotics and enhanced cell membrane permeability occur following HMO exposure (21, 25, 26). In addition, HMO exposure perturbs multiple GBS metabolic pathways including those related to linoleic acid, sphingolipid, glycerophospholipid, and pyrimidine metabolism (26). A transposon mutant library screen identified the *gbs0738* gene (locus *san*_0913 or GBSCOH1_RS04065 in COH1), a putative glycosyltransferase family 8 protein, as essential for GBS susceptibility to HMOs over a 7-h time course (21); however, the functional role of this glycosyltransferase in GBS-host interactions and resistance to antimicrobial compounds has not been characterized. Using a targeted insertional mutant of *san*_0913 (COH1 Δ*san*_0913) (21), we assessed the growth of wild-type (WT) COH1 and Δ*san*_0913 in the presence of 0 to 20 mg/mL pHMOs over 18 h. We found that growth of COH1 was significantly inhibited at all pHMO concentrations tested compared to the mock control (Fig. 2A and B). Concentrations of 20 and 10 mg/mL pHMO inhibited growth of Δ*san*_0913 but to a lesser degree than seen with COH1 (Fig. 2A and B). To determine whether *san*_0913 disruption altered GBS characteristics associated with colonization, we assessed the ability of Δ*san*_0913 to form biofilms and attach to vaginal epithelial cells. We observed no differences between COH1 and Δ*san*_0913 biofilm formation in either bacteriologic (Todd-Hewitt broth [THB]) or eukaryotic (RPMI 1640) media, as measured by crystal violet staining (Fig. 2C). In addition, we observed no differences in VK2 adherence between the COH1 and Δ*san*_0913 strains (Fig. 2D). In our *in vivo* model, we found no differences in vaginal GBS burdens between COH1 and Δ*san*_0913 (Fig. 2E). However, when mice were treated with pHMOs as in Fig. 1A, Δ*san*_0913 displayed significantly higher GBS burdens at day 1 postinoculation (*P = *0.007) during active pHMO treatment, but this difference resolved at later time points (Fig. 2F). Furthermore, we performed MIC assays of a variety of antibiotic classes, hydrogen peroxide, and dimethyl sulfoxide (DMSO) to determine the impact of *san*_0913 deficiency on GBS susceptibility. MICs were determined by a >90% reduction in control optical density at 600 nm (OD_600_) values. No differences in MICs between COH1 and Δ*san*_0913 were observed with any compound tested (see Table S1 in the supplemental material).

10.1128/msphere.00885-21.1 TABLE S1 Minimum inhibitory concentrations for WT COH1 and Δ*san_*0913. All experiments were done in at least three independent experiments in technical duplicate. Data were analyzed by Wilcoxon rank test with median values and confidence interval (CI 95%) calculated from the MICs of each independent experiment. Download Table S1, DOCX file, 0.01 MB. Copyright © 2022 Mejia et al. 2022 Mejia et al. https://creativecommons.org/licenses/by/4.0/ This content is distributed under the terms of the Creative Commons Attribution 4.0 International license.

### pHMO treatment minimally impacts the endogenous murine vaginal microbiota in the presence or absence of GBS.

**FIG 3:**
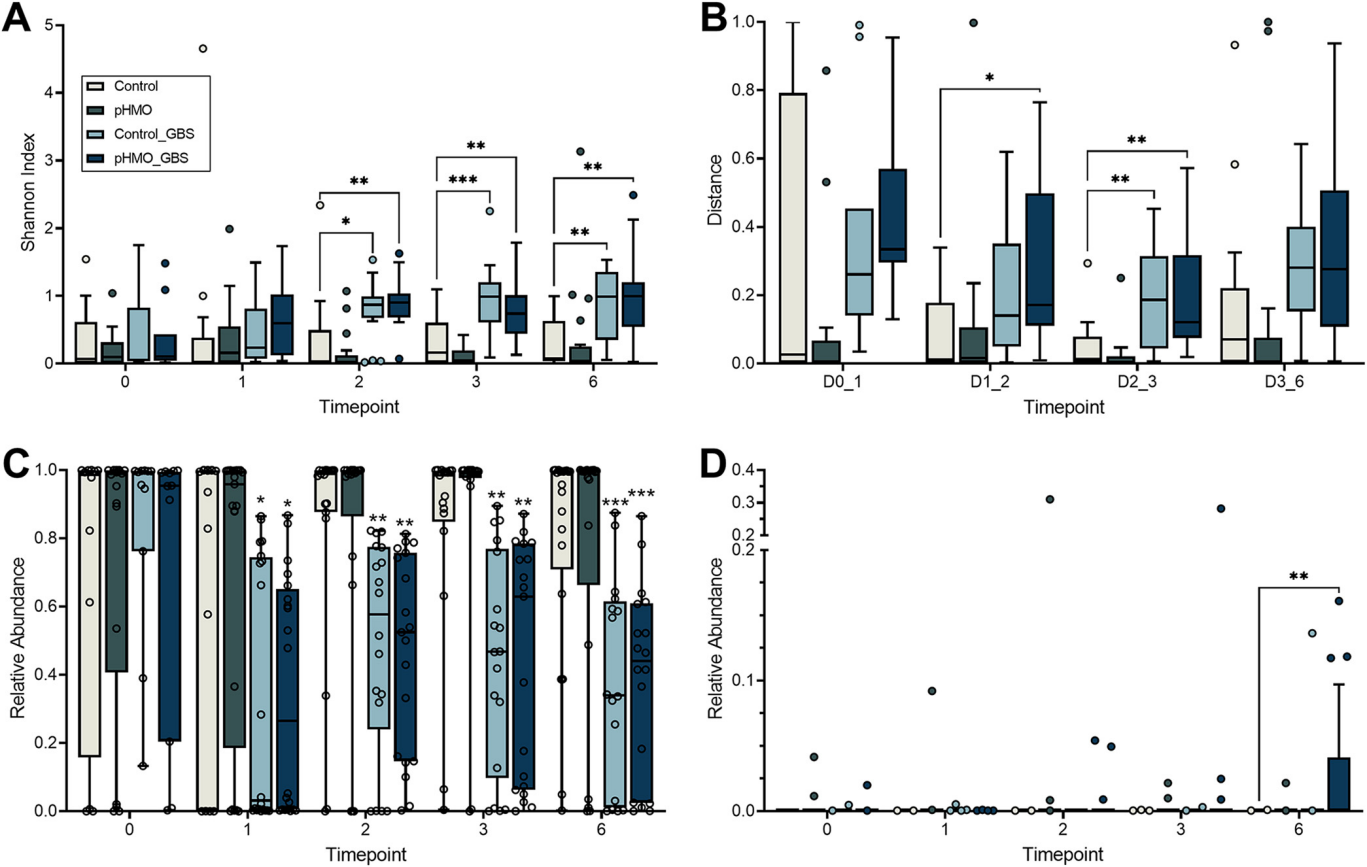
Alpha and beta diversity and differential taxa abundance, as measured by 16S rRNA amplicon sequencing. Mice were mock infected or GBS infected and then treated with pHMOs or mock treated: Control (mock-treated, mock-infected), pHMO (treated, mock-infected), Control_GBS (mock-treated, GBS-infected), and pHMO_GBS (treated, GBS-infected) as described in Materials and Methods. (A) Shannon’s diversity index of vaginal 16S amplicon sequencing from each condition over the time course. (B) Bray-Curtis pairwise distances between subsequent time points. (C and D) Relative abundances of *S. succinus* (C) and *Bacteroides* spp. (D) according to treatment group over time. Results are displayed as a Tukey’s box plot (A, B, and D) and min-to-max box-and-whisker plots (C) (*n *= 11 to 21/group per time point). Data were analyzed by two-way repeated measures ANOVA with Tukey’s multiple-comparison test. All comparisons shown are versus the Control group. ***, *P < *0.001; **, *P < *0.01; *, *P < *0.05. All other comparisons are not significant.

We previously identified that GBS introduction to the murine vaginal tract causes community instability, particularly a decrease in Staphylococcus succinus, a dominant vaginal microbe in C57BL/6J mice (33). Because HMOs are metabolized by a variety of bacteria in the neonatal intestines (34–38), and since maternal serum HMO levels correlate with specific taxa in the maternal urinary and vaginal microbiota (39), we investigated whether pHMO treatment impacted the murine vaginal microbiota in the presence or absence of GBS perturbation. Using swabs from the murine experiments outlined in Fig. 1A, 16S rRNA amplicon sequencing was used to characterize shifts in the vaginal microbiota of Control (mock-treated, mock-infected), pHMO (treated, mock-infected), Control_GBS (mock-treated, GBS-infected), and pHMO_GBS (treated, GBS-infected) mice. Alpha diversity, as measured by Shannon’s diversity index, significantly increased in Control_GBS and pHMO_GBS groups compared to controls (Fig. 3A). However, in the absence of GBS, alpha diversity was not impacted in the pHMO versus control groups at any time point (Fig. 3A). As observed previously (33), mice that received GBS showed heightened community instability compared to mock-infected controls as measured by Bray-Curtis distance between time points. This effect was seen both in the presence (pHMO_GBS, *P = *0.0048) and absence (Control_GBS, *P = *0.0073) of pHMO treatment for pairwise comparisons between days 2 and 3 (Fig. 3B). No impact on community stability was observed with pHMO treatment in the absence of GBS (pHMO, Fig. 3B).

Across all four conditions, no significant differences were observed in community richness over the 6-day time course, as measured by observed operational taxonomic units (OTUs; see Fig. S2A). Mice exposed to GBS (Control_GBS and pHMO_GBS), regardless of treatment, experienced a significant drop in the relative abundance of *S. succinus* compared to Control mice starting at day 1, and this effect continued throughout the sampling period (Fig. 3C). No differences in the relative abundance of *Enterococcus* spp. or *Lactobacillus* spp., the two next most abundant endogenous OTUs, were observed between groups (see Fig. S2B and C). ANCOM analysis (40) identified *Bacteroides* as the only significant differentially abundant taxa across the four groups, with increased abundance in pHMO_GBS mice compared to all other groups (Fig. 3D).

10.1128/msphere.00885-21.3 FIG S2 Richness and relative abundances of vaginal 16S samples. Mice were mock-infected or GBS-infected and treated with pHMOs or mock treatment: Control (mock-treated, mock-infected), pHMO (treated, mock-infected), Control_GBS (mock-treated, GBS-infected), and pHMO_GBS (treated, GBS-infected), as described in Materials and Methods. (A) Observed OTUs in mouse samples across the four experimental conditions over time. (B and C) Relative abundances of *Enterococcus* spp. (B) and *Lactobacillus* spp. (C) over time. All graphs are displayed as Tukey’s box plots. Data were analyzed by two-way repeated measures ANOVA with Tukey’s multiple-comparison test. All comparisons are not significant. Download FIG S2, TIF file, 0.2 MB. Copyright © 2022 Mejia et al. 2022 Mejia et al. https://creativecommons.org/licenses/by/4.0/ This content is distributed under the terms of the Creative Commons Attribution 4.0 International license.

### Murine vaginal community state types display minimal differential stability upon pHMO treatment in the presence or absence of GBS.

**FIG 4:**
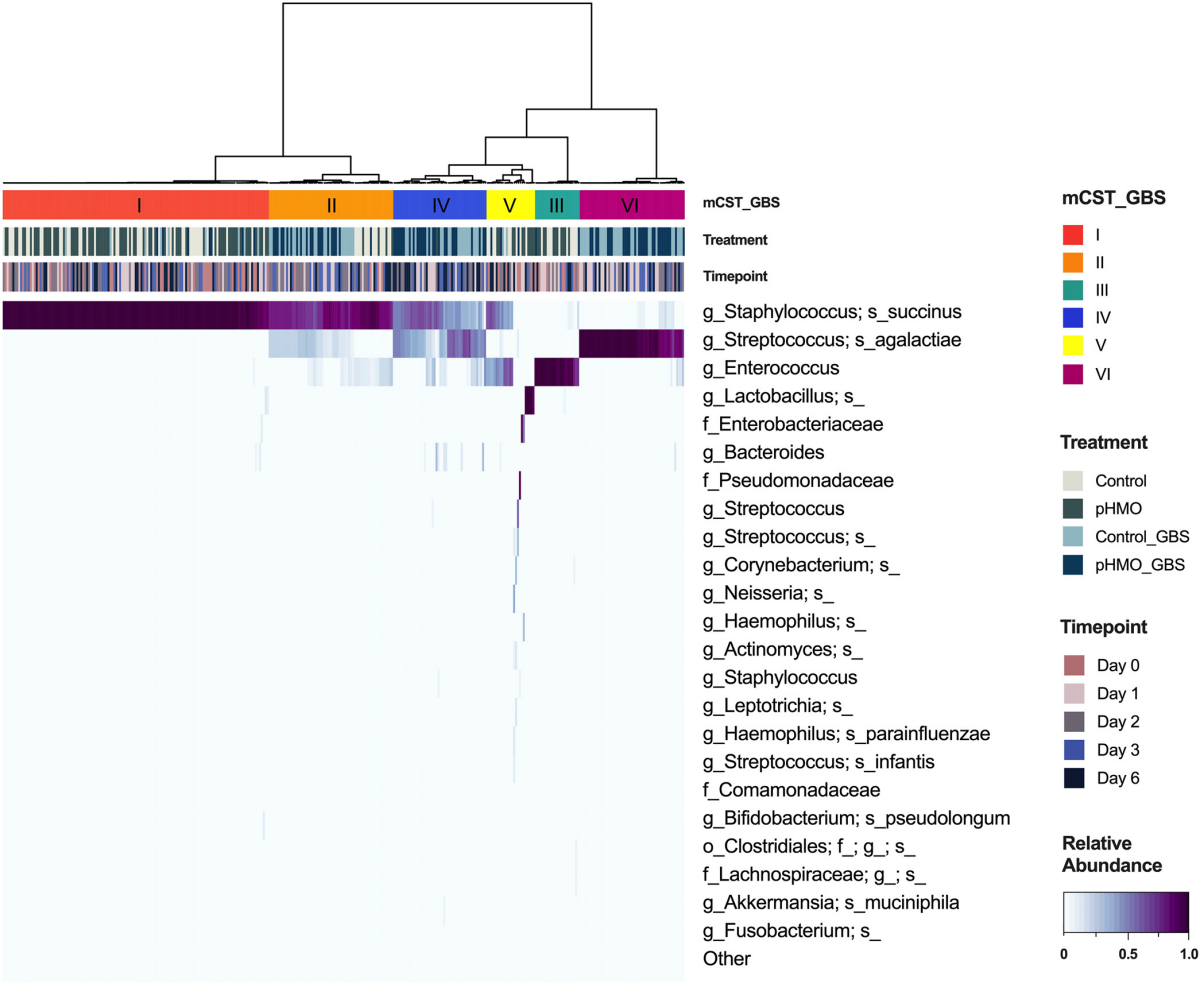
Heatmap of murine community state types across treatment groups and time points. The relative abundances of the top 23 taxa in mice across all four treatment groups as determined by 16S rRNA amplicon sequencing (*n *=* *20 to 24 mice/group) are shown. Murine samples are hierarchically clustered by Ward’s linkage of Euclidean distances to generate mCSTs (top bar). The treatment (middle bar) and time point (bottom bar) per sample are displayed above the heatmap. Highest to lowest taxonomic abundances are indicated by heatmap intensity corresponding to the color bar (indicated in lower right corner), ranging from dark purple to white.

The human vaginal microbiome, and more recently the murine vaginal microbiome, are classified into community state types (CSTs) (41) and murine community state types (mCSTs), respectively (33). In humans, four CSTs are each dominated by different *Lactobacillus* species, and the remaining CST had a non-*Lactobacillus* dominant taxa or diverse array of facultative and strictly anaerobic bacteria (41). In C57BL/6J mice from Jackson Laboratory, the vaginal microbiome is separated into 5 mCSTs dominated by either *S. succinus*, *Enterococcus*, a *S. succinus*-*Enterococcus* mixture, *Lactobacillus*, or a mixture of different taxa (33). In the present study, we detected all five of these mCSTs by hierarchical clustering with Ward’s linkage of Euclidean distances of day 0 swab samples prior to GBS infection and/or pHMO treatment (see Fig. S3). When analyzing samples from all four groups across all time points, we observed the emergence of three GBS-containing groups: (i) GBS dominant (mCST VI), (ii) GBS and *S. succinus* present at similar levels (mCST IV), and (iii) *S. succinus* dominant with lower abundances of GBS or *Enterococcus* (mCST II) (Fig. 4).

10.1128/msphere.00885-21.4 FIG S3 Baseline mCST clustering of C57BL/6J mouse samples from this study combined with our prior study. Vaginal swab samples taken prior to treatment (day 0) or from mock controls (uninfected, untreated) were hierarchically clustered by Ward’s linkage of Euclidean distances to generate mCSTs (top bar). The relative abundances of the top 34 taxa are displayed in a heatmap where the highest to lowest taxonomic abundances correspond to the color bar (indicated in the upper left corner), ranging from dark purple to white. Data were combined from the present study and data deposited at EBI under accession number PRJEB25733 (33). Download FIG S3, TIF file, 1.4 MB. Copyright © 2022 Mejia et al. 2022 Mejia et al. https://creativecommons.org/licenses/by/4.0/ This content is distributed under the terms of the Creative Commons Attribution 4.0 International license.

**FIG 5:**
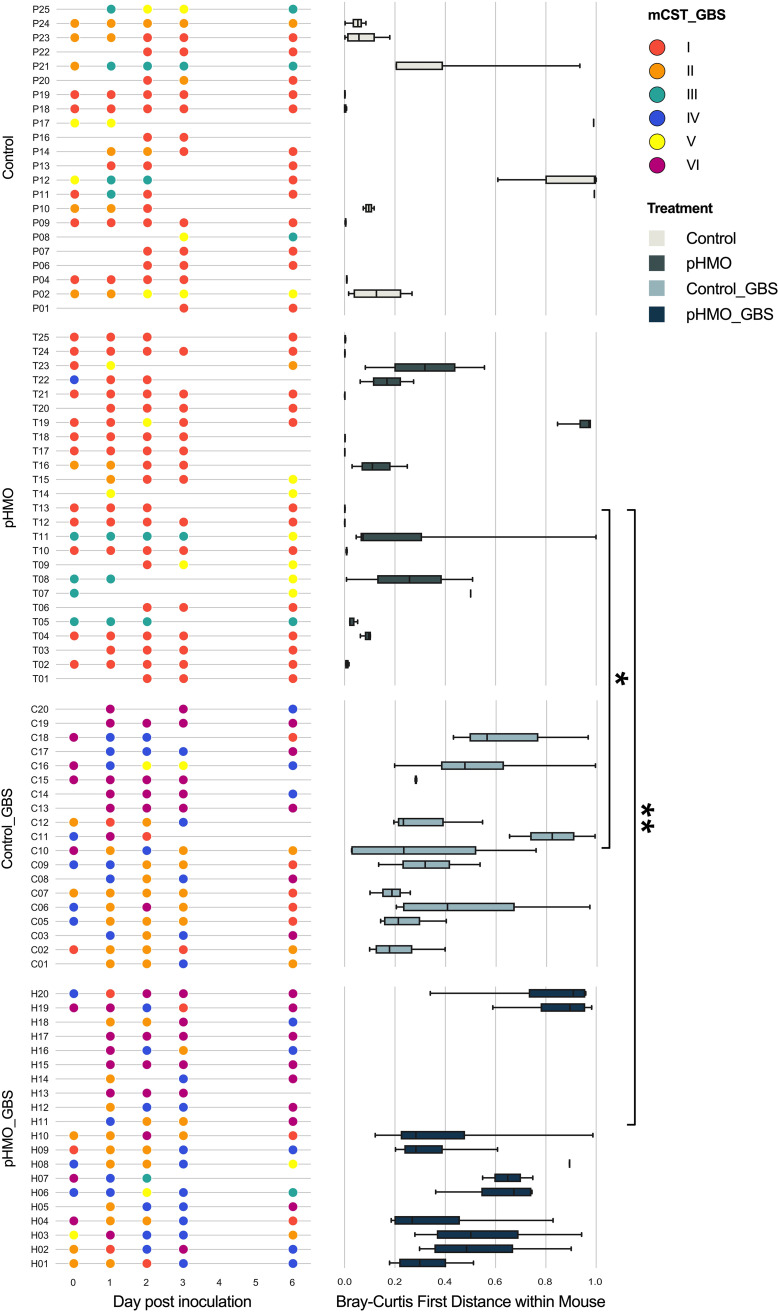
Vaginal microbiome stability over time with pHMO treatment and/or GBS infection. mCST designations for mouse cohort samples are displayed ordered by treatment group and time point (left panels). For each mouse, corresponding Bray-Curtis first distances from the day zero time point are shown (right panels). Mice with fewer than two sequenced samples were excluded from analysis and mice without a sequenced day 0 sample were excluded from the first distance analysis (*n *=* *20 to 25/group). Data were analyzed by Kruskal-Wallis test with a Dunn’s multiple-comparison posttest. **, *P < *0.01; *, *P < *0.05. All other comparisons are not significant.

To assess whether mice differentially transitioned between mCSTs among treatment groups, we tracked mCSTs in individual mice over time. As our prior study (33), we found that mCSTs were relative unstable, with 43% of uninfected and 87% of GBS-infected mice categorized to two or more mCSTs over the time course (Fig. 5). Using Bray-Curtis first distances for microbial communities within individual mice, we compared the instability between the baseline composition and the subsequent time points. Although there were no differences in longitudinal stability between Control and pHMO groups (*P *= 0.9615) or Control and Control_GBS groups (*P* > 0.9999), Bray-Curtis first distances were higher in Control_GBS versus pHMO (*P *= 0.042) and pHMO_GBS versus pHMO mice (*P *= 0.0003; Fig. 5).

**FIG 6:**
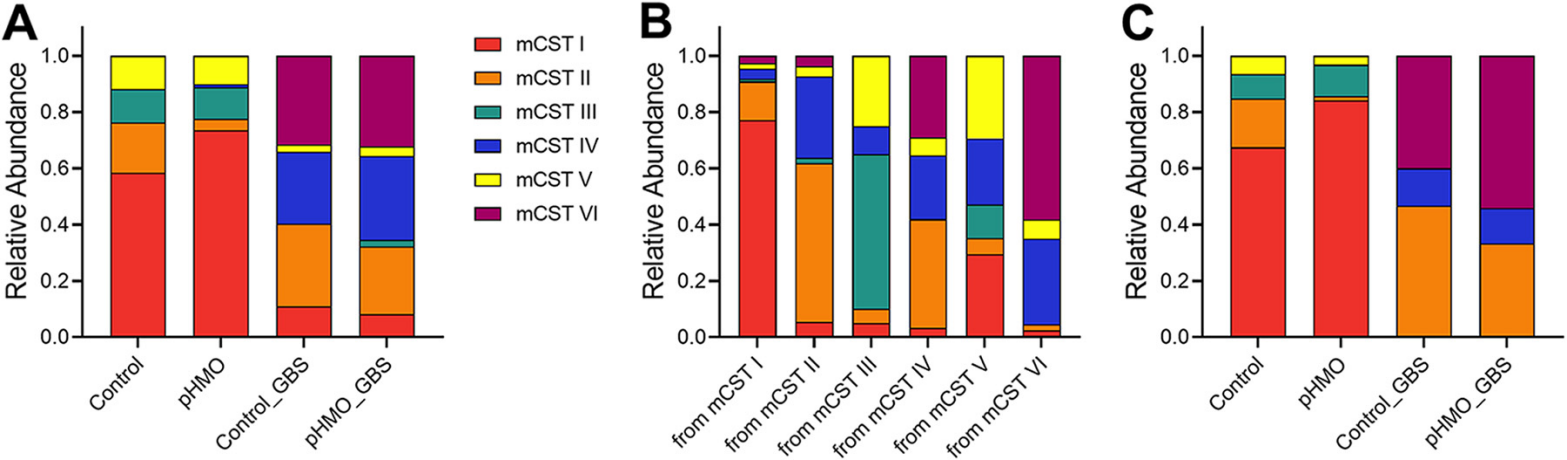
Frequency and transitions of mCSTs across treatment groups. mCST designations for mouse cohort samples were combined from all time points. (A) Frequency of mCST appearances within treatment groups. (B) Proportion of samples designated to each mCST grouped by the mCST from the previous time point. A self-transitioning mCST would be designated from a mCST to the same mCST at the next time point (e.g., from mCST I to mCST I). (C) Relative proportions of mCSTs that self-transitioned at the next time point separated by treatment group. Data were analyzed by chi-square test.

Although mCST I (*S. succinus*-dominant) was the most common mCST in Control and pHMO groups, mCST II appeared with significantly more frequency in the Control group (*P* = 0.0404) and mCST I appeared with more frequency in the pHMO group (*P *= 0.0067) (Fig. 6A). No significant differences in mCST frequencies were observed between Control_GBS and pHMO_groups with mCST II, mCST IV, and mCST VI representing the most abundant mCSTs in both GBS-infected groups (Fig. 6A). As seen previously (33), mCST I was the most stable community state: combining all conditions and samples with successfully sequenced consecutive time points, 84/109 (77%) of mCST I samples were assigned mCST I at the next time point (self-transitioning). mCST VI (GBS-dominant) was the next most stable, followed by mCST II, mCST III, mCST V, and mCST IV (Fig. 6B). When separated by treatment groups, we found that mCST I was more likely to self-transition in the pHMO group compared to the Control group (*P *= 0.0401), whereas mCST II was more likely to self-transition in the Control group compared to the pHMO group (*P *= 0.0031) (Fig. 6C). In GBS-infected animals, no significant differences in mCST self-transitions were observed between Control_GBS and pHMO_GBS (Fig. 6C).

## DISCUSSION

GBS remains a pervasive pathogen in pregnancy and the neonatal period. Current IAP prevention strategies have not fully abolished GBS neonatal infections and IAP is ineffective in preventing GBS infection prior to parturition. Because of the adverse effects of antibiotic exposure on the endogenous microbiota and propagation of antibiotic resistance, discovery of more targeted antimicrobial therapies to control maternal GBS carriage is important for maternal and neonatal health. Here, we apply HMOs, natural products produced by the mammary gland during pregnancy and lactation to *in vitro* and murine models of GBS vaginal colonization. HMOs are known for simultaneous prebiotic benefits on commensal bacteria (14, 34, 38) and antimicrobial activity toward pathogens, including GBS (21–24). To our knowledge, this is the first application of HMOs as a vaginal therapy *in vivo*. We propose that HMOs possess promising anti-GBS activity in this environment with minimal impact on the vaginal microbiota.

Our animal model demonstrated that pHMO treatment reduced GBS vaginal carriage, but this effect was only seen during active treatment with no sustained impact observed after treatment ceased (Fig. 1). This finding aligns with other murine models showing protective effects of HMOs in reducing pathogen colonization (31, 42–44). We observed no changes in bacterial adherence when VK2 cells were pretreated with pHMOs. This observation is distinct from work showing HMO-mediated inhibition of pathogens (31, 45–47) or enhanced attachment of beneficial bacteria (32, 48–50) at the gastrointestinal mucosa. Other studies have observed no impact of pHMO treatment on certain pathogens (51) or on pathogen colonization of other epithelial surfaces such as the bladder (52). These results suggest that prior mechanisms seen with HMOs and the gut epithelium may absent in the vaginal epithelium or with the bacterial species we tested.

There are several limitations to this HMO treatment model. First, we did not optimize dosage, timing, or length of pHMO treatment, nor did we assess the impact of HMO treatment or *san*_0913 deficiency across multiple GBS strain or serotypes. Pilot studies failed to show an effect of a 0.3-mg HMO dose using the same treatment regimen applied in Fig. 1A (data not shown); however, it is possible that dosages greater than 1 mg used here may provide more significant reduction of GBS burden or alterations to the vaginal microbiota. Second, although LNT shows potent *in vitro* anti-GBS activity (21), this did not translate to an *in vivo* GBS reduction, and thus the specific HMOs responsible for GBS reduction in our animal model are currently unknown. A clinical study found that Lewis positive women, who generate certain fucosylated HMOs, display reduced GBS vaginal carriage and infant colonization at birth (27). Specifically, levels of lacto-N-difucohexaose I (LNDFHI) in breastmilk samples negatively correlated with maternal GBS colonization status and reduced GBS growth *in vitro* (27). Successful identification of the specific HMO(s) providing activity *in vivo* is a critical future step to translate our findings to the clinic. Third, HMOs and their fermentation products have multiple known gastrointestinal epithelial and immune modulatory activities (53–56). Likewise, it is possible that HMOs act indirectly through altering host vaginal responses to GBS; however, this was not evaluated in our study. Lastly, using murine models to test whether HMOs possess potential therapeutic activity in preventing GBS neonatal transmission and adverse birth outcomes (57, 58) will be an important application of our findings.

Although the exact mechanism of anti-GBS activity by HMOs is unknown, GBS susceptibility is linked to expression of a GBS-specific putative glycosyltransferase (locus *san*_0913) thought to catalyze the addition of glucose or galactose residues to the cell surface and thus may enable incorporation of HMOs into the GBS cell wall (21). In prior work, a glycosyltransferase-deficient Δ*san*_0913 strain showed resistance to HMO inhibition (5 mg/mL) over 7 h of culture (21). In our growth analysis, we confirmed this finding extended out to 18 h (Fig. 2). At higher concentrations (10 to 20 mg/mL) matching physiologic concentration of HMOs in human colostrum and breastmilk (59, 60), Δ*san*_0913 growth was inhibited, but not to the same extent as COH1, suggesting that this deficiency does not completely resolve anti-GBS activity of HMOs. Recent work has shown that HMOs induced multiple GBS stress responses related to cell membrane and cell wall components (26), but the role of *san*_0913 in this GBS response has not been established. While streptococcal glycosyltransferase activity has been implicated in biofilm formation and composition in S. mutans (61), our phenotypic analyses did not reveal any substantial deficits in the glycosyltransferase-deficient Δ*san*_0913 in biofilm formation, vaginal cell adherence, or *in vivo* vaginal colonization in the absence of HMO treatment. In the presence of HMOs, *san*_0913 deficiency conferred a colonization advantage early on during colonization, but not at later time points, suggesting that direct inhibition by HMOs at least partially contributes to HMO-mediated GBS reduction *in vivo*. These results may have important clinical implications for HMO therapies and emergence of spontaneous HMO-resistant GBS under selective pressure.

HMOs serve as prebiotics in the gut by promoting the establishment of *Bifidobacteria* and *Bacteroides* (37, 38, 62). Mammary HMO production begins early in pregnancy and is detected in maternal circulation in the first trimester (63). Moreover, maternal serum levels of two abundant HMOs (2′-FL and 3′-SL) positively correlate with vaginal *Gardnerella* spp. and L. crispatus, respectively (39), providing a basis for the hypothesis that HMOs might not only shape neonatal microbiota and immunity but also maternal vaginal microbiota. Whether HMOs have the potential to directly impact the vaginal microbiome in humans has not been determined, however, a common vaginal species, L. gasseri, lacks the ability to metabolize HMOs (34). Because of the well-known prebiotic effects of HMOs on the infant microbiota, we examined the impact of pHMOs on the murine vaginal microbiota in our colonization model. We found minimal pHMO-driven changes to the community composition in terms of alpha and beta diversity (Fig. 3). The most marked difference between groups in our model was the emergence of *Bacteroides* in mice dually inoculated with GBS and treated with pHMOs (Fig. 3D). While the relative abundance of *Bacteroides* remained below 5% of the entire microbial landscape in the majority of mice, 0.1 to 5% abundance is estimated to account for ca. 10^5^ to 10^6^ total CFU in the murine vaginal tract. In women, the vaginal microbiota postpartum shows community instability and increases in *Bifidobacterium* and *Bacteroides* (64, 65), but the mechanisms driving these changes are unknown. Whether HMOs can be detected in the human vagina during pregnancy and lactation, and whether human vaginal microbes can metabolize HMOs are important topics of future study.

There are several limitations to the interpretation of our murine microbiome data. First and foremost, the murine vaginal microbiome does not fully reflect the human vaginal microbiome in terms of species present; although there is an mCST dominated by a murine *Lactobacillus* (see Fig. S2), it is a rare community in C57BL/6J mice (33). As a future direction, we seek to use humanized microbiota mice to assess pHMO-mediated changes to the vaginal microbiota in the presence of human vaginal bacteria, such as that done in mouse models colonized with human gastrointestinal microbiota and treated with HMOs (42, 66). In women, GBS is present at a low relative abundance in the vagina (67), whereas in our mouse model GBS becomes a dominant member of vaginal community in some mice upon introduction (Fig. 4). This high relative abundance may alter dynamics of GBS and other vaginal taxa distinct from human vaginal communities. In addition, the length of HMO treatment may need to be extended to observe larger effects. Prior studies have described more pronounced HMO-mediated shifts to the gut microbiota of both conventional (44, 68) and humanized microbiota mice (66); however, the length of HMO treatment in these studies was longer than in our model (3 to 8 weeks versus 3 days, respectively).

By combining our prior (33) and current studies, we found that the vaginal microbiome of the C57BL/6J mice from one vendor is highly consistent across cohorts over several years. In both studies, we found that GBS introduction increases vaginal community instability and reduces the relative abundance of the most abundant taxa *S. succinus*. In addition, we confirmed our prior observation that mCST I (*S. succinus* dominant) is the most stable murine community over time. These consistencies highlight the utility of this murine model in comparing different experimental groups across cohorts and experimental variables.

In summary, we have demonstrated HMOs can reduce GBS vaginal colonization in an animal model with minimal impacts on the vaginal microbiota. There is mounting evidence that HMOs play an important role in shaping the infant gut microbiota and preventing pathogen colonization. HMO introduction to the vaginal tract may provide similar beneficial effects. These findings lay the framework to expand our knowledge of therapeutic applications of HMOs and support their continued development as a target for controlling GBS colonization in women.

## MATERIALS AND METHODS

### Reagents, bacterial strains, and cell lines.

Pooled HMOs were isolated from human milk samples collected through a donation program at UC San Diego, lyophilized, and stored at −20°C as described previously (69). Individual HMO lacto-N-tetraose (LNT) was purchased from Dextra Laboratories. Prior to use, HMOs were resuspended in molecular grade water to a final concentration of 100 mg/ml, and subsequent dilutions were made in cell culture media (*in vitro*) or molecular grade water (*in vivo*).

Group B Streptococcus (GBS) strains COH1 (ATCC BAA-1176) and isogenic Δ*san*_0913 generated previously (21) were grown for at least 16 h at 37°C in Todd-Hewitt Broth (THB) prior to experiments with 5 μg/mL erythromycin added to Δ*san*_0913 cultures. Prior to *in vitro* and *in vivo* experiments, overnight cultures were diluted 1:10 in fresh THB and incubated stationary at 37°C until mid-log phase (OD_600_* *= 0.4). Lactobacillus rhamnosus GG (ATCC 53103) was grown for 16 h at 37°C without shaking in de Man, Rogosa, and Sharpe (MRS) broth.

Immortalized human vaginal epithelial cells (VK2/E6E7, ATCC CRL-2616) were cultured in keratinocyte serum-free medium (KSFM) (Gibco) with 0.5 ng/mL human recombinant epidermal growth factor and 0.05 mg/mL bovine pituitary extract. Cells were cultured in a 37°C incubator with 5% CO_2_. Cells were split every 3 to 4 days at ∼80% confluence, and 0.25% trypsin–2.21 mM EDTA (Corning) was used to detach cells for passaging.

### GBS growth kinetics.

For growth curves, log phase cultures were diluted 1:10 in RPMI 1640 (Gibco) in 96-well microtiter plates with 20, 10, 5, or 2.5 mg/mL pHMOs or water control in a 200-μL total volume. Wells with pHMOs and media only were included to confirm absence of microbial contamination. Plates were incubated at 37°C, and the absorbance at OD_600_ was read every 15 min for 18 h using a BioTek Cytation 5 multimode plate reader.

### Biofilm assays.

GBS biofilm assays were performed as described previously (70). Briefly, overnight cultures were diluted to an OD_600_ of 0.1 in RPMI 1640 or THB, followed by incubation at 37°C for 24 h. The medium was removed, and the biofilms were washed twice with phosphate-buffered saline (PBS) before drying at 55°C for 30 min. Biofilms were stained with 0.2% crystal violet for 30 min, washed with PBS three times, and destained with 80:20 ethanol-acetone. Supernatant was transferred to a fresh 96-well plate, and the absorbance was measured at OD_595_ using a BioTek Cytation 5 plate reader. Values were normalized to total bacterial growth prior to washing and staining, and data were expressed as a ratio of crystal violet staining to total bacterial growth (OD_595_ to OD_600_).

### MIC assays.

MICs were performed as described with minor adaptions (71). Mid-log-phase cultures were diluted 1:100 in THB with or without H_2_O_2_, DMSO (Fisher Scientific), trimethoprim (Sigma), chloramphenicol (Fisher Scientific), and vancomycin (Sigma) at concentrations listed in Table S1 in a 100-μL total volume in 96-well microtiter plates. Plates were incubated stationary for 24 h at 37°C. The MICs were determined by a >90% reduction in OD_600_ absorbance compared to control wells.

### Adherence assays.

GBS adherence assays were performed on confluent VK2 cells in 24-well plates as described previously (72, 73). For studies using HMOs, medium was replaced with KSFM containing 3 or 6 mg/mL pHMO, LNT, or vehicle control for 18 h. Cells were infected with GBS COH1, Δ*san*_0913, or L. rhamnosus at MOI = 1 (assuming 1 × 10^5^ VK2 cells per well). Bacteria were brought into contact with the VK2 cells by centrifugation for 1 min at 300 × *g*. After 30 min, the supernatant was removed, and the cells were washed six times with sterile PBS. Cell layers were incubated for 5 min with 100 μL of 0.25% trypsin–2.21 mM EDTA, and then 400 μL of 0.025% Triton-X in PBS was added. Wells were mixed 30 times to ensure detachment, and bacterial recovery was determined by plating on THB or MRS agar plates using serial dilution and counting CFU. Data are expressed as the percentage of adherent CFU compared to the original inoculum.

### Animals.

Animal experiments were approved by the UC San Diego and Baylor College of Medicine Institutional Animal Care and Use Committees (IACUC) and conducted under accepted veterinary standards. Mice were allowed to eat and drink *ad libitum*. WT C57BL/6J female mice, originally purchased from Jackson Laboratories, aged 7 weeks, were allowed to acclimate for 1 week prior to experiments.

### Murine GBS vaginal colonization model.

Vaginal colonization studies were conducted as described previously (74). Briefly, mice were synchronized with 0.5 mg of β-estradiol administered intraperitoneally 24 h prior to inoculation. Mice were inoculated with 10 μL (1 × 10^7^ CFU total) of GBS COH1 or PBS as a mock control into the vaginal tract. Where applicable, mice were administered 1 mg (10 μL of 100 mg/mL) pHMOs, LNT, or vehicle control into the vaginal lumen at 2 h postinoculation. Vaginal swabs were collected daily, about 24 h apart. In experiments testing HMOs, mouse received additional HMO or mock treatments on days 1 and 2 immediately after swab collection. Recovered GBS (identified as pink/mauve colonies) was quantified by plating on CHROMagar StrepB (DRG International, Inc.). Growth of blue colonies was considered endogenous *Enterococcus* spp. based on manufacturer protocols. Remaining swab samples were stored at −20°C until further use.

### Sample processing and 16S rRNA amplicon sequencing.

DNA was extracted from thawed bacterial swab suspensions using a Quick-DNA Fungal/Bacterial Microprep kit protocol (Zymo Research). The V4 regions of the 16S rRNA gene were amplified using barcoded 515F-806R primers (75), and the resulting V4 amplicons were sequenced on an Illumina MiSeq. Raw sequencing data were transferred to Qiita (76). Sequences were demultiplexed, trimmed to 150-bp reads, and denoised using Deblur through QIIME2 v2020.8 (77). Qiime2 was also used for rarefaction (1,900 sequences per sample) and calculation of the alpha diversity (Shannon and OTUs) and beta diversity (Bray-Curtis distance). For ANCOM (40) analysis for differentially abundant OTUs, the nonrarefied feature table was used. Taxonomic assignments used the naive Bayes sklearn classifier in QIIME 2 trained on the 515F/806R region of Greengenes 13_8 99% OTUs. Since many of the samples were low biomass, DNA contaminants from sequencing reagents and kits had a substantial impact on the data set. Negative controls that went through the entire pipeline, from DNA extraction to sequencing, were used to catalog these contaminants (Pseudomonas veronii). Mitochondria and chloroplast 16S sequences were also removed. Output files generated through the Qiime2 pipeline were exported and analyzed with R version 3.6.1 (2019-07-05; “Action of the Toes”) using stats, factoextra, and Phyloseq (78, 79). Data visualization was performed with ggplot2 (80) and Seaborn (81).

### Community state type delineation.

Feature tables and representative sequences generated from three individual studies were merged and used to generate a taxonomy file. Two more studies from our prior work (33) were downloaded from EBI accession number PRJEB25733 in addition to the present study (EBI accession PRJEB49304) for Fig. S2 depicting the Baseline CSTs. To assign mCSTs and create heatmaps, hierarchical clustering was performed using the R package stats (79) on the rarefied feature table with Ward’s linkage of Euclidean distances. The optimum number of clusters (5 mCSTs) was determined using wss and silhouette (kmeans) based on the dendrogram. For EBI accession number PRJEB49304 (this study) alone, including all experimental conditions and time points, we added an additional GBS-dominant mCST, as modeled previously (33). For within-mouse assessment of instability and mCST transitioning, samples with only one time point collected were excluded. Samples that did not successfully sequence at the baseline (day 0) time point were excluded from Bray-Curtis first distances analysis.

### Statistics.

All data were collected from at least three biological replicates performed in at least technical duplicate as part of at least two independent experiments. When biological replicates were not available (e.g., immortalized cell lines and bacteria only assays), experiments were performed independently at least three times. Mean values from technical replicates were used for statistical analyses, with independent experiment values or biological replicates represented in graphs with means, medians with interquartile ranges, or box-and-whisker plots with Tukey’s, as indicated in the figure legends. All data sets were subjected to the D’Agostino and Pearson normality test to determine whether values displayed Gaussian distribution before selecting the appropriate parametric or nonparametric analyses. In instances where *in vitro* and *in vivo* experimental *n* values were too small to determine normality, the data were assumed to be nonparametric. GBS vaginal colonization burdens were assessed using Kruskal-Wallis with Dunn’s multiple-comparison test or two-stage Mann-Whitney test, as indicated in figure legends. GBS adherence to VK2 cells was assessed by or two-way analysis of variance (ANOVA) with Dunnett’s multiple-comparison test or the Wilcoxon matched-pairs signed rank test, as indicated in figure legends. GBS growth (area under curve) and biofilm formation were compared using two-way repeated measures ANOVA with Dunnett’s multiple-comparison test and two-way ANOVA with Sidak’s multiple-comparison test, respectively. Data from 16S rRNA amplicon sequencing were analyzed by two-way ANOVA with Tukey’s comparison. Bray-Curtis first distances were analyzed by using a Kruskal-Wallis test with a Dunn’s multiple-comparison posttest. mCST transition frequencies were compared by using a chi-square test. Statistical analyses were performed using GraphPad Prism, version 9.2.0 (GraphPad Software, Inc., La Jolla, CA). *P* values of <0.05 were considered statistically significant.

### Data availability.

Sequencing Data used in this study is available in EBI under accession number PRJEB49304; the code is accessible at GitHub under project “HMO_GBScategorization.”

## References

[1] Russell NJ, Seale AC, O’Driscoll M, O’Sullivan C, Bianchi-Jassir F, Gonzalez-Guarin J, Lawn JE, Baker CJ, Bartlett L, Cutland C, Gravett MG, Heath PT, Le Doare K, Madhi SA, Rubens CE, Schrag S, Sobanjo-Ter Meulen A, Vekemans J, Saha SK, Ip M, GBS Maternal Colonization Investigator Group. 2017. Maternal colonization with group B streptococcus and serotype distribution worldwide: systematic review and meta-analyses. Clin Infect Dis 65:S100–S111. doi:10.1093/cid/cix658. 29117327 PMC5848259

[2] Seale AC, Bianchi-Jassir F, Russell NJ, Kohli-Lynch M, Tann CJ, Hall J, Madrid L, Blencowe H, Cousens S, Baker CJ, Bartlett L, Cutland C, Gravett MG, Heath PT, Ip M, Le Doare K, Madhi SA, Rubens CE, Saha SK, Schrag SJ, Sobanjo-Ter Meulen A, Vekemans J, Lawn JE. 2017. Estimates of the burden of group B streptococcal disease worldwide for pregnant women, stillbirths, and children. Clin Infect Dis 65:S200–S219. doi:10.1093/cid/cix664. 29117332 PMC5849940

[3] Le Doare K, Heath PT. 2013. An overview of global GBS epidemiology. Vaccine 31(Suppl 4):D7–D12. doi:10.1016/j.vaccine.2013.01.009. 23973349

[4] McDonald HM, Chambers HM. 2000. Intrauterine infection and spontaneous midgestation abortion: is the spectrum of microorganisms similar to that in preterm labor? Infect Dis Obstet Gynecol 8:220–227. doi:10.1155/S1064744900000314. 11220481 PMC1784699

[5] Phares CR, Lynfield R, Farley MM, Mohle-Boetani J, Harrison LH, Petit S, Craig AS, Schaffner W, Zansky SM, Gershman K, Stefonek KR, Albanese BA, Zell ER, Schuchat A, Schrag SJ, Active Bacterial Core surveillance/Emerging Infections Program Network. 2008. Epidemiology of invasive group B streptococcal disease in the United States, 1999–2005. JAMA 299:2056–2065. doi:10.1001/jama.299.17.2056. 18460666

[6] Azad MB, Konya T, Persaud RR, Guttman DS, Chari RS, Field CJ, Sears MR, Mandhane PJ, Turvey SE, Subbarao P, Becker AB, Scott JA, Kozyrskyj AL, Investigators CS, CHILD Study Investigators. 2016. Impact of maternal intrapartum antibiotics, method of birth and breastfeeding on gut microbiota during the first year of life: a prospective cohort study. BJOG 123:983–993. doi:10.1111/1471-0528.13601. 26412384

[7] Mazzola G, Murphy K, Ross RP, Di Gioia D, Biavati B, Corvaglia LT, Faldella G, Stanton C. 2016. Early gut microbiota perturbations following intrapartum antibiotic prophylaxis to prevent group B streptococcal disease. PLoS One 11:e0157527. doi:10.1371/journal.pone.0157527. 27332552 PMC4917232

[8] Parnanen K, Karkman A, Hultman J, Lyra C, Bengtsson-Palme J, Larsson DGJ, Rautava S, Isolauri E, Salminen S, Kumar H, Satokari R, Virta M. 2018. Maternal gut and breast milk microbiota affect infant gut antibiotic resistome and mobile genetic elements. Nat Commun 9:3891. doi:10.1038/s41467-018-06393-w. 30250208 PMC6155145

[9] Li H, Xiao B, Zhang Y, Xiao S, Luo J, Huang W. 2019. Impact of maternal intrapartum antibiotics on the initial oral microbiome of neonates. Pediatr Neonatol 60:654–661. doi:10.1016/j.pedneo.2019.03.011. 31056339

[10] Patras KA, Nizet V. 2018. Group B streptococcal maternal colonization and neonatal disease: molecular mechanisms and preventative approaches. Front Pediatr 6:27. doi:10.3389/fped.2018.00027. 29520354 PMC5827363

[11] Frank NM, Lynch KF, Uusitalo U, Yang J, Lonnrot M, Virtanen SM, Hyoty H, Norris JM, Group TS, TEDDY Study Group. 2019. The relationship between breastfeeding and reported respiratory and gastrointestinal infection rates in young children. BMC Pediatr 19:339. doi:10.1186/s12887-019-1693-2. 31533753 PMC6749679

[12] Stewart CJ, Ajami NJ, O’Brien JL, Hutchinson DS, Smith DP, Wong MC, Ross MC, Lloyd RE, Doddapaneni H, Metcalf GA, Muzny D, Gibbs RA, Vatanen T, Huttenhower C, Xavier RJ, Rewers M, Hagopian W, Toppari J, Ziegler AG, She JX, Akolkar B, Lernmark A, Hyoty H, Vehik K, Krischer JP, Petrosino JF. 2018. Temporal development of the gut microbiome in early childhood from the TEDDY study. Nature 562:583–588. doi:10.1038/s41586-018-0617-x. 30356187 PMC6415775

[13] Donovan SM, Comstock SS. 2016. Human milk oligosaccharides influence neonatal mucosal and systemic immunity. Ann Nutr Metab 69(Suppl 2):42–51. doi:10.1159/000452818. 28103609 PMC6392703

[14] Sela DA, Chapman J, Adeuya A, Kim JH, Chen F, Whitehead TR, Lapidus A, Rokhsar DS, Lebrilla CB, German JB, Price NP, Richardson PM, Mills DA. 2008. The genome sequence of *Bifidobacterium longum* subsp. *infantis* reveals adaptations for milk utilization within the infant microbiome. Proc Natl Acad Sci USA 105:18964–18969. doi:10.1073/pnas.0809584105. 19033196 PMC2596198

[15] Bode L. 2012. Human milk oligosaccharides: every baby needs a sugar mama. Glycobiology 22:1147–1162. doi:10.1093/glycob/cws074. 22513036 PMC3406618

[16] Triantis V, Bode L, van Neerven RJJ. 2018. Immunological effects of human milk oligosaccharides. Front Pediatr 6:190. doi:10.3389/fped.2018.00190. 30013961 PMC6036705

[17] Newburg DS. 2009. Neonatal protection by an innate immune system of human milk consisting of oligosaccharides and glycans. J Anim Sci 87:26–34. doi:10.2527/jas.2008-1347. 19028867

[18] Laucirica DR, Triantis V, Schoemaker R, Estes MK, Ramani S. 2017. Milk oligosaccharides inhibit human rotavirus infectivity in MA104 cells. J Nutr 147:1709–1714. 28637685 10.3945/jn.116.246090 PMC5572490

[19] Newburg DS, Pickering LK, McCluer RH, Cleary TG. 1990. Fucosylated oligosaccharides of human milk protect suckling mice from heat-stabile enterotoxin of *Escherichia coli*. J Infect Dis 162:1075–1080. doi:10.1093/infdis/162.5.1075. 2230234

[20] Idota T, Kawakami H, Murakami Y, Sugawara M. 1995. Inhibition of cholera toxin by human milk fractions and sialyllactose. Biosci Biotechnol Biochem 59:417–419. doi:10.1271/bbb.59.417. 7766178

[21] Lin AE, Autran CA, Szyszka A, Escajadillo T, Huang M, Godula K, Prudden AR, Boons GJ, Lewis AL, Doran KS, Nizet V, Bode L. 2017. Human milk oligosaccharides inhibit growth of group B *Streptococcus*. J Biol Chem 292:11243–11249. doi:10.1074/jbc.M117.789974. 28416607 PMC5500792

[22] Ackerman DL, Doster RS, Weitkamp JH, Aronoff DM, Gaddy JA, Townsend SD. 2017. Human milk oligosaccharides exhibit antimicrobial and antibiofilm properties against group B *Streptococcus*. ACS Infect Dis 3:595–605. doi:10.1021/acsinfecdis.7b00064. 28570820 PMC5868341

[23] Craft KM, Thomas HC, Townsend SD. 2019. Sialylated variants of lacto-N-tetraose exhibit antimicrobial activity against group B *Streptococcus*. Org Biomol Chem 17:1893–1900. doi:10.1039/c8ob02080a. 30229793

[24] Craft KM, Thomas HC, Townsend SD. 2018. Interrogation of human milk oligosaccharide fucosylation patterns for antimicrobial and antibiofilm trends in group B *Streptococcus*. ACS Infect Dis 4:1755–1765. doi:10.1021/acsinfecdis.8b00234. 30350565

[25] Craft KM, Gaddy JA, Townsend SD. 2018. Human milk oligosaccharides (HMOs) sensitize group B streptococcus to clindamycin, erythromycin, gentamicin, and minocycline on a strain specific basis. ACS Chem Biol 13:2020–2026. doi:10.1021/acschembio.8b00661. 30071726

[26] Chambers SA, Moore RE, Craft KM, Thomas HC, Das R, Manning SD, Codreanu SG, Sherrod SD, Aronoff DM, McLean JA, Gaddy JA, Townsend SD. 2020. A solution to antifolate resistance in group B *Streptococcus*: untargeted metabolomics identifies human milk oligosaccharide-induced perturbations that result in potentiation of trimethoprim. mBio 11:e00076-20. doi:10.1128/mBio.00076-20. 32184236 PMC7078465

[27] Andreas NJ, Al-Khalidi A, Jaiteh M, Clarke E, Hyde MJ, Modi N, Holmes E, Kampmann B, Mehring Le Doare K. 2016. Role of human milk oligosaccharides in group B *Streptococcus* colonization. Clin Trans Immunol 5:e99. doi:10.1038/cti.2016.43. PMC5007626 27588204

[28] Wilson CB, Weaver WM. 1985. Comparative susceptibility of group B streptococci and *Staphylococcus aureus* to killing by oxygen metabolites. J Infect Dis 152:323–329. doi:10.1093/infdis/152.2.323. 2993435

[29] Chen P, Reiter T, Huang B, Kong N, Weimer BC. 2017. Prebiotic oligosaccharides potentiate host protective responses against *L. monocytogenes* infection. Pathogens 6:68. doi:10.3390/pathogens6040068. PMC5750592 29257110

[30] Coppa GV, Zampini L, Galeazzi T, Facinelli B, Ferrante L, Capretti R, Orazio G. 2006. Human milk oligosaccharides inhibit the adhesion to Caco-2 cells of diarrheal pathogens: *Escherichia coli*, *Vibrio cholerae*, and *Salmonella fyris*. Pediatr Res 59:377–382. doi:10.1203/01.pdr.0000200805.45593.17. 16492975

[31] Manthey CF, Autran CA, Eckmann L, Bode L. 2014. Human milk oligosaccharides protect against enteropathogenic *Escherichia coli* attachment *in vitro* and EPEC colonization in suckling mice. J Pediatr Gastroenterol Nutr 58:165–168. doi:10.1097/MPG.0000000000000172. 24048169 PMC8865036

[32] Kavanaugh DW, O’Callaghan J, Butto LF, Slattery H, Lane J, Clyne M, Kane M, Joshi L, Hickey RM. 2013. Exposure of *Bifidobacterium longum* subsp. *infantis* to milk oligosaccharides increases adhesion to epithelial cells and induces a substantial transcriptional response. PLoS One 8:e67224. doi:10.1371/journal.pone.0067224. 23805302 PMC3689703

[33] Vrbanac A, Riestra AM, Coady A, Knight R, Nizet V, Patras KA. 2018. The murine vaginal microbiota and its perturbation by the human pathogen group B streptococcus. BMC Microbiol 18:197. doi:10.1186/s12866-018-1341-2. 30477439 PMC6260558

[34] Ward RE, Ninonuevo M, Mills DA, Lebrilla CB, German JB. 2006. *In vitro* fermentation of breast milk oligosaccharides by *Bifidobacterium infantis* and *Lactobacillus gasseri*. Appl Environ Microbiol 72:4497–4499. doi:10.1128/AEM.02515-05. 16751577 PMC1489581

[35] Underwood MA, Gaerlan S, De Leoz ML, Dimapasoc L, Kalanetra KM, Lemay DG, German JB, Mills DA, Lebrilla CB. 2015. Human milk oligosaccharides in premature infants: absorption, excretion, and influence on the intestinal microbiota. Pediatr Res 78:670–677. doi:10.1038/pr.2015.162. 26322410 PMC4689671

[36] Sakanaka M, Gotoh A, Yoshida K, Odamaki T, Koguchi H, Xiao JZ, Kitaoka M, Katayama T. 2019. Varied pathways of infant gut-associated bifidobacterium to assimilate human milk oligosaccharides: prevalence of the gene set and its correlation with bifidobacterium-rich microbiota formation. Nutrients 12:71. doi:10.3390/nu12010071. PMC7019425 31888048

[37] Marcobal A, Barboza M, Sonnenburg ED, Pudlo N, Martens EC, Desai P, Lebrilla CB, Weimer BC, Mills DA, German JB, Sonnenburg JL. 2011. Bacteroides in the infant gut consume milk oligosaccharides via mucus-utilization pathways. Cell Host Microbe 10:507–514. doi:10.1016/j.chom.2011.10.007. 22036470 PMC3227561

[38] Yu ZT, Chen C, Newburg DS. 2013. Utilization of major fucosylated and sialylated human milk oligosaccharides by isolated human gut microbes. Glycobiology 23:1281–1292. doi:10.1093/glycob/cwt065. 24013960 PMC3796377

[39] Pausan MR, Kolovetsiou-Kreiner V, Richter GL, Madl T, Giselbrecht E, Obermayer-Pietsch B, Weiss EC, Jantscher-Krenn E, Moissl-Eichinger C. 2020. Human milk oligosaccharides modulate the risk for preterm birth in a microbiome-dependent and -independent manner. mSystems 5:e00324-20. doi:10.1128/mSystems.00334-20. PMC7289590 32518196

[40] Mandal S, Van Treuren W, White RA, Eggesbo M, Knight R, Peddada SD. 2015. Analysis of composition of microbiomes: a novel method for studying microbial composition. Microb Ecol Health Dis 26:27663. doi:10.3402/mehd.v26.27663. 26028277 PMC4450248

[41] France MT, Ma B, Gajer P, Brown S, Humphrys MS, Holm JB, Waetjen LE, Brotman RM, Ravel J. 2020. VALENCIA: a nearest centroid classification method for vaginal microbial communities based on composition. Microbiome 8:166. doi:10.1186/s40168-020-00934-6. 33228810 PMC7684964

[42] Musilova S, Modrackova N, Hermanova P, Hudcovic T, Svejstil R, Rada V, Tejnecky V, Bunesova V. 2017. Assessment of the synbiotic properties of human milk oligosaccharides and *Bifidobacterium longum* subsp. *infantis in vitro* and in humanized mice. Benef Microbes 8:281–289. doi:10.3920/BM2016.0138. 28116928

[43] Yu ZT, Nanthakumar NN, Newburg DS. 2016. The human milk oligosaccharide 2′-fucosyllactose quenches *Campylobacter jejuni*-induced inflammation in human epithelial cells HEp-2 and HT-29 and in mouse intestinal mucosa. J Nutr 146:1980–1990. doi:10.3945/jn.116.230706. 27629573 PMC5037868

[44] Wang Y, Zou Y, Wang J, Ma H, Zhang B, Wang S. 2020. The protective effects of 2′-fucosyllactose against *Escherichia coli* O157 infection are mediated by the regulation of gut microbiota and the inhibition of pathogen adhesion. Nutrients 12:1284. doi:10.3390/nu12051284. PMC7282266 32369957

[45] Wu RY, Li B, Koike Y, Maattanen P, Miyake H, Cadete M, Johnson-Henry KC, Botts SR, Lee C, Abrahamsson TR, Landberg E, Pierro A, Sherman PM. 2019. Human milk oligosaccharides increase mucin expression in experimental necrotizing enterocolitis. Mol Nutr Food Res 63:e1800658. doi:10.1002/mnfr.201800658. 30407734

[46] Ruiz-Palacios GM, Cervantes LE, Ramos P, Chavez-Munguia B, Newburg DS. 2003. *Campylobacter jejuni* binds intestinal H(O) antigen (Fuc-α-1, 2Gal-β1, 4GlcNAc) and fucosyloligosaccharides of human milk inhibit its binding and infection. J Biol Chem 278:14112–14120. doi:10.1074/jbc.M207744200. 12562767

[47] Weichert S, Jennewein S, Hufner E, Weiss C, Borkowski J, Putze J, Schroten H. 2013. Bioengineered 2′-fucosyllactose and 3-fucosyllactose inhibit the adhesion of *Pseudomonas aeruginosa* and enteric pathogens to human intestinal and respiratory cell lines. Nutr Res 33:831–838. doi:10.1016/j.nutres.2013.07.009. 24074741

[48] Quinn EM, Slattery H, Thompson AP, Kilcoyne M, Joshi L, Hickey RM. 2018. Mining milk for factors which increase the adherence of *Bifidobacterium longum* subsp. *infantis* to intestinal cells. Foods 7:196. doi:10.3390/foods7120196. PMC6306836 30513877

[49] Chichlowski M, De Lartigue G, German JB, Raybould HE, Mills DA. 2012. Bifidobacteria isolated from infants and cultured on human milk oligosaccharides affect intestinal epithelial function. J Pediatr Gastroenterol Nutr 55:321–327. doi:10.1097/MPG.0b013e31824fb899. 22383026 PMC3381975

[50] Zhang G, Zhao J, Wen R, Zhu X, Liu L, Li C. 2020. 2′-Fucosyllactose promotes *Bifidobacterium bifidum* DNG6 adhesion to Caco-2 cells. J Dairy Sci 103:9825–9834. doi:10.3168/jds.2020-18773. 32896399

[51] Facinelli B, Marini E, Magi G, Zampini L, Santoro L, Catassi C, Monachesi C, Gabrielli O, Coppa GV. 2019. Breast milk oligosaccharides: effects of 2′-fucosyllactose and 6′-sialyllactose on the adhesion of *Escherichia coli* and *Salmonella fyris* to Caco-2 cells. J Matern Fetal Neonatal Med 32:2950–2952. doi:10.1080/14767058.2018.1450864. 29562795

[52] Lin AE, Autran CA, Espanola SD, Bode L, Nizet V. 2014. Human milk oligosaccharides protect bladder epithelial cells against uropathogenic *Escherichia coli* invasion and cytotoxicity. J Infect Dis 209:389–398. doi:10.1093/infdis/jit464. 23990566 PMC3883170

[53] Fukuda S, Toh H, Hase K, Oshima K, Nakanishi Y, Yoshimura K, Tobe T, Clarke JM, Topping DL, Suzuki T, Taylor TD, Itoh K, Kikuchi J, Morita H, Hattori M, Ohno H. 2011. Bifidobacteria can protect from enteropathogenic infection through production of acetate. Nature 469:543–547. doi:10.1038/nature09646. 21270894

[54] Salli K, Anglenius H, Hirvonen J, Hibberd AA, Ahonen I, Saarinen MT, Tiihonen K, Maukonen J, Ouwehand AC. 2019. The effect of 2′-fucosyllactose on simulated infant gut microbiome and metabolites; a pilot study in comparison to GOS and lactose. Sci Rep 9:13232. doi:10.1038/s41598-019-49497-z. 31520068 PMC6744565

[55] He Y, Liu S, Kling DE, Leone S, Lawlor NT, Huang Y, Feinberg SB, Hill DR, Newburg DS. 2016. The human milk oligosaccharide 2′-fucosyllactose modulates CD14 expression in human enterocytes, thereby attenuating LPS-induced inflammation. Gut 65:33–46. doi:10.1136/gutjnl-2014-307544. 25431457

[56] Xiao L, van De Worp WR, Stassen R, van Maastrigt C, Kettelarij N, Stahl B, Blijenberg B, Overbeek SA, Folkerts G, Garssen J, Van’t Land B. 2019. Human milk oligosaccharides promote immune tolerance via direct interactions with human dendritic cells. Eur J Immunol 49:1001–1014. doi:10.1002/eji.201847971. 30900752 PMC6619030

[57] Andrade EB, Magalhaes A, Puga A, Costa M, Bravo J, Portugal CC, Ribeiro A, Correia-Neves M, Faustino A, Firon A, Trieu-Cuot P, Summavielle T, Ferreira P. 2018. A mouse model reproducing the pathophysiology of neonatal group B streptococcal infection. Nat Commun 9:3138. doi:10.1038/s41467-018-05492-y. 30087335 PMC6081475

[58] Randis TM, Gelber SE, Hooven TA, Abellar RG, Akabas LH, Lewis EL, Walker LB, Byland LM, Nizet V, Ratner AJ. 2014. Group B streptococcus beta-hemolysin/cytolysin breaches maternal-fetal barriers to cause preterm birth and intrauterine fetal demise *in vivo*. J Infect Dis 210:265–273. doi:10.1093/infdis/jiu067. 24474814 PMC4092248

[59] Thurl S, Munzert M, Boehm G, Matthews C, Stahl B. 2017. Systematic review of the concentrations of oligosaccharides in human milk. Nutr Rev 75:920–933. doi:10.1093/nutrit/nux044. 29053807 PMC5914348

[60] Soyyılmaz B, Mikš MH, Röhrig CH, Matwiejuk M, Meszaros-Matwiejuk A, Vigsnæs LK. 2021. The mean of milk: a review of human milk oligosaccharide concentrations throughout lactation. Nutrients 13:2737. doi:10.3390/nu13082737. 34444897 PMC8398195

[61] Rainey K, Michalek SM, Wen ZT, Wu H. 2019. Glycosyltransferase-mediated biofilm matrix dynamics and virulence of *Streptococcus mutans*. Appl Environ Microbiol 85:e00247-18. doi:10.1128/AEM.02247-18. 30578260 PMC6384114

[62] Davis EC, Wang M, Donovan SM. 2017. The role of early life nutrition in the establishment of gastrointestinal microbial composition and function. Gut Microbes 8:143–171. doi:10.1080/19490976.2016.1278104. 28068209 PMC5390825

[63] Jantscher-Krenn E, Aigner J, Reiter B, Kofeler H, Csapo B, Desoye G, Bode L, van Poppel MNM. 2019. Evidence of human milk oligosaccharides in maternal circulation already during pregnancy: a pilot study. Am J Physiol Endocrinol Metab 316:E347–E357. doi:10.1152/ajpendo.00320.2018. 30422706

[64] MacIntyre DA, Chandiramani M, Lee YS, Kindinger L, Smith A, Angelopoulos N, Lehne B, Arulkumaran S, Brown R, Teoh TG, Holmes E, Nicoholson JK, Marchesi JR, Bennett PR. 2015. The vaginal microbiome during pregnancy and the postpartum period in a European population. Sci Rep 5:8988. doi:10.1038/srep08988. 25758319 PMC4355684

[65] Freitas AC, Hill JE. 2018. Bifidobacteria isolated from vaginal and gut microbiomes are indistinguishable by comparative genomics. PLoS One 13:e0196290. doi:10.1371/journal.pone.0196290. 29684056 PMC5912743

[66] Rubio-Del-Campo A, Gozalbo-Rovira R, Moya-Gonzalvez EM, Alberola J, Rodriguez-Diaz J, Yebra MJ. 2021. Infant gut microbiota modulation by human milk disaccharides in humanized microbiome mice. Gut Microbes 13:1–20. doi:10.1080/19490976.2021.1914377. PMC8096338 33938391

[67] Pace RM, Chu DM, Prince AL, Ma J, Seferovic MD, Aagaard KM. 2021. Complex species and strain ecology of the vaginal microbiome from pregnancy to postpartum and association with preterm birth. Medicine 2:1027–1049. doi:10.1016/j.medj.2021.06.001. PMC8491999 34617072

[68] Arnold JW, Whittington HD, Dagher SF, Roach J, Azcarate-Peril MA, Bruno-Barcena JM. 2021. Safety and modulatory effects of humanized galacto-oligosaccharides on the gut microbiome. Front Nutr 8:640100. doi:10.3389/fnut.2021.640100. 33898497 PMC8058378

[69] Jantscher-Krenn E, Zherebtsov M, Nissan C, Goth K, Guner YS, Naidu N, Choudhury B, Grishin AV, Ford HR, Bode L. 2012. The human milk oligosaccharide disialyllacto-*N*-tetraose prevents necrotizing enterocolitis in neonatal rats. Gut 61:1417–1425. doi:10.1136/gutjnl-2011-301404. 22138535 PMC3909680

[70] Patras KA, Derieux J, Al-Bassam MM, Adiletta N, Vrbanac A, Lapek JD, Zengler K, Gonzalez DJ, Nizet V. 2018. Group B streptococcus biofilm regulatory protein A contributes to bacterial physiology and innate immune resistance. J Infect Dis 218:1641–1652. doi:10.1093/infdis/jiy341. 29868829 PMC6173572

[71] Patras KA, Coady A, Babu P, Shing SR, Ha AD, Rooholfada E, Brandt SL, Geriak M, Gallo RL, Nizet V. 2020. Host cathelicidin exacerbates group B streptococcus urinary tract infection. mSphere 5:e00932-19. doi:10.1128/mSphere.00932-19. PMC7178553 32321824

[72] Lum GR, Mercado V, van Ens D, Nizet V, Kimmey JM, Patras KA. 2021. Hypoxia-inducible factor 1 alpha is dispensable for host defense of group B streptococcus colonization and infection. J Innate Immun 13:391–403. doi:10.1159/000515739. 34023827 PMC8613573

[73] Patras KA, Wescombe PA, Rosler B, Hale JD, Tagg JR, Doran KS. 2015. *Streptococcus salivarius* K-12 limits group B streptococcus vaginal colonization. Infect Immun 83:3438–3444. doi:10.1128/IAI.00409-15. 26077762 PMC4534663

[74] Patras KA, Doran KS. 2016. A murine model of group B streptococcus vaginal colonization. J Vis Exp 2016:54708. doi:10.3791/54708. PMC5226234 27911391

[75] Caporaso JG, Lauber CL, Walters WA, Berg-Lyons D, Lozupone CA, Turnbaugh PJ, Fierer N, Knight R. 2011. Global patterns of 16S rRNA diversity at a depth of millions of sequences per sample. Proc Natl Acad Sci USA 108(Suppl 1):4516–4522. doi:10.1073/pnas.1000080107. 20534432 PMC3063599

[76] Gonzalez A, Navas-Molina JA, Kosciolek T, McDonald D, Vazquez-Baeza Y, Ackermann G, DeReus J, Janssen S, Swafford AD, Orchanian SB, Sanders JG, Shorenstein J, Holste H, Petrus S, Robbins-Pianka A, Brislawn CJ, Wang M, Rideout JR, Bolyen E, Dillon M, Caporaso JG, Dorrestein PC, Knight R. 2018. Qiita: rapid, web-enabled microbiome meta-analysis. Nat Methods 15:796–798. doi:10.1038/s41592-018-0141-9. 30275573 PMC6235622

[77] Bolyen E, Rideout JR, Dillon MR, Bokulich NA, Abnet CC, Al-Ghalith GA, Alexander H, Alm EJ, Arumugam M, Asnicar F, Bai Y, Bisanz JE, Bittinger K, Brejnrod A, Brislawn CJ, Brown CT, Callahan BJ, Caraballo-Rodríguez AM, Chase J, Cope EK, Da Silva R, Diener C, Dorrestein PC, Douglas GM, Durall DM, Duvallet C, Edwardson CF, Ernst M, Estaki M, Fouquier J, Gauglitz JM, Gibbons SM, Gibson DL, Gonzalez A, Gorlick K, Guo J, Hillmann B, Holmes S, Holste H, Huttenhower C, Huttley GA, Janssen S, Jarmusch AK, Jiang L, Kaehler BD, Kang KB, Keefe CR, Keim P, Kelley ST, Knights D, et al. 2019. Reproducible, interactive, scalable, and extensible microbiome data science using QIIME 2. Nat Biotechnol 37:852–857. doi:10.1038/s41587-019-0209-9. 31341288 PMC7015180

[78] McMurdie PJ, Holmes S. 2013. phyloseq: an R package for reproducible interactive analysis and graphics of microbiome census data. PLoS One 8:e61217. doi:10.1371/journal.pone.0061217. 23630581 PMC3632530

[79] R Core Team. 2013. R: a language and environment for statistical computing. R Foundation for Statistical Computing, Vienna, Austria. http://www.R-project.org/.

[80] Wickham H. 2016. ggplot2: elegant graphics for data analysis. Springer-Verlag, New York, NY.

[81] Waskom M. 2021. Seaborn: statistical data visualization. Joss 6:3021. doi:10.21105/joss.03021.

