# Supplementary figures and images for: Human Milk Oligosaccharides Reduce Murine Group B Streptococcus Vaginal Colonization with Minimal Impact on the Vaginal Microbiota
Source: mSphere. 2022 Jan 5;7(1):e00885-21. doi: 10.1128/msphere.00885-21 (PMC8730812; doi:10.1128/msphere.00885-21)

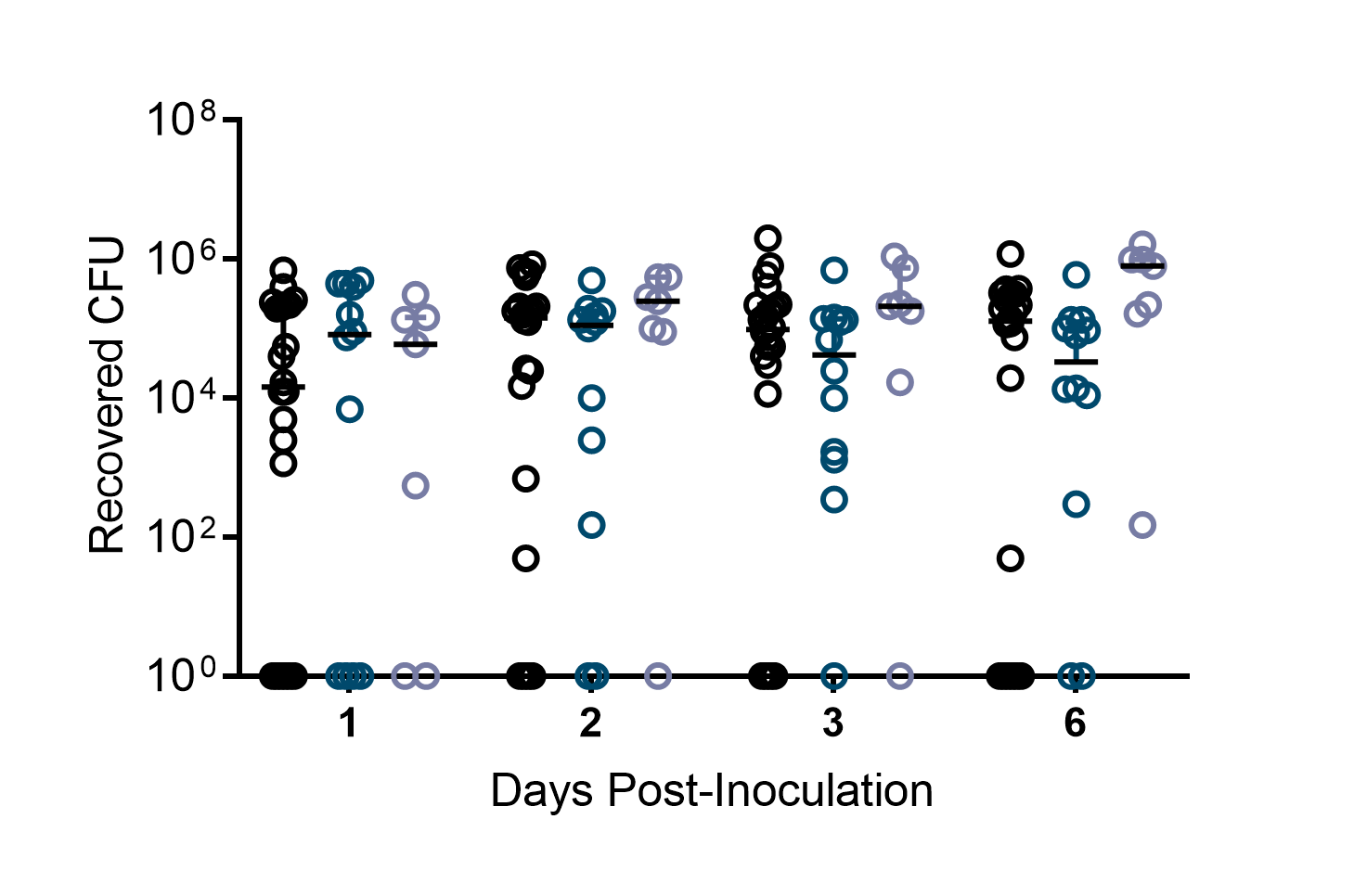

Supplement: FIG S1 [file msphere.00885-21-sf001.tif]

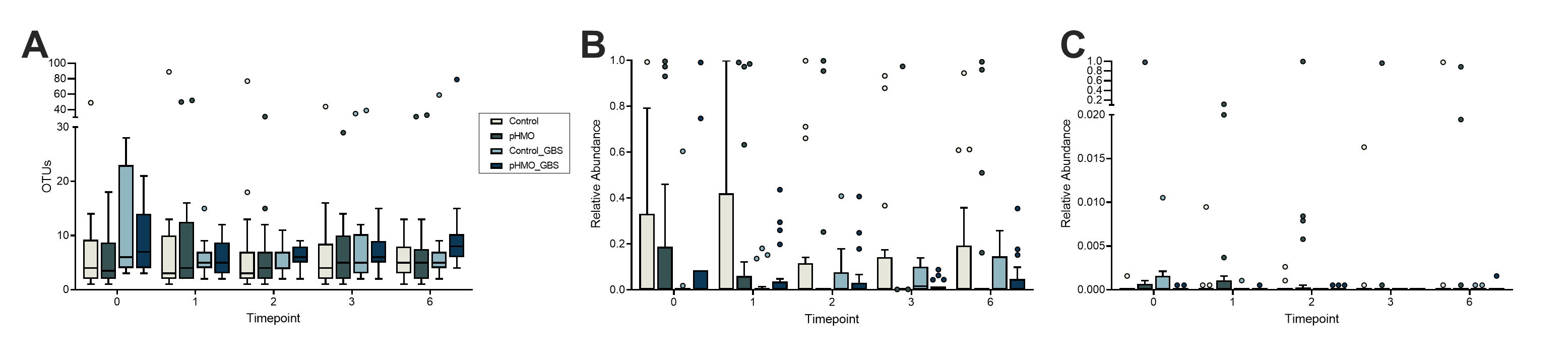

Supplement: FIG S2 [file msphere.00885-21-sf002.tif]

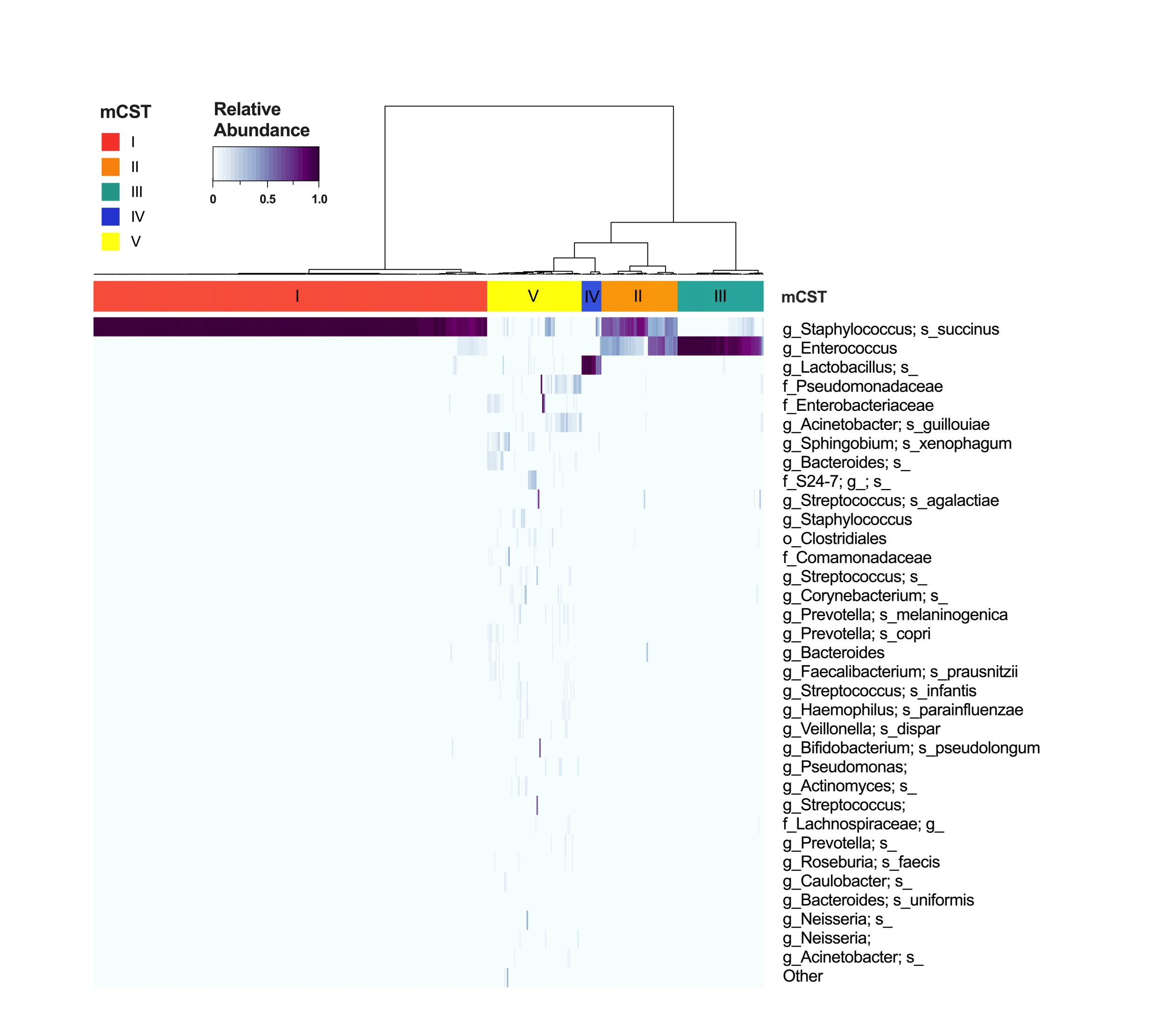

Supplement: FIG S3 [file msphere.00885-21-sf003.tif]
