# Supplementary material for: Human Milk Oligosaccharides Reduce Murine Group B Streptococcus Vaginal Colonization with Minimal Impact on the Vaginal Microbiota
Source: mSphere. 2022 Jan 5;7(1):e00885-21. doi: 10.1128/msphere.00885-21 (PMC8730812; doi:10.1128/msphere.00885-21)
Supplement: TABLE S1 [file msphere.00885-21-st001.docx]

**Supplemental Table 1**

|  | **WT COH1** (CI 95%) | **Δ*san_*0913** (CI 95%) | Concentrations tested |
| --- | --- | --- | --- |
| Chloramphenicol | **1.25** (1.25-2.5) | **2.5** (1.25-2.5) | 0.625 – 40 µg/mL |
| DMSO | **20** (20) | **20** (10-20) | 0.31 – 80% (v/v) |
| H_2_O_2_ | **0.0093** (0.0093) | **0.0093** (0.0093) | 0.0047 – 0.3% (v/v) |
| Trimethoprim | **0.3125** (0.125-5) | **0.15625** (0.125-0.25) | 0.0078 – 20 mg/mL |
| Vancomycin | **0.125** (0.125-1) | **0.125** (0.125-1) | 0.031 – 2 µg/mL |
